# Optical Coherence Tomography Angiography for Evaluation of Conjunctival Vessels in Dry Eyes

**DOI:** 10.1155/2023/1609332

**Published:** 2023-10-14

**Authors:** TongFeng Cui, HongYan Sun, ZiZhong Hu, YaBo Shi, Jiang Zhu, ManMan Jin, Bing Qin

**Affiliations:** ^1^Department of Ophthalmology, Suqian First Hospital, Suqian, China; ^2^Department of Ophthalmology, The First Affiliated Hospital of Nanjing Medical University, Nanjing, China

## Abstract

**Objective:**

This study aimed to evaluate conjunctival vessels in patients with dry eye disease (DED) using optical coherence tomography angiography (OCTA).

**Methods:**

This was a cross-sectional, observational clinical study. Twenty-three eyes of 18 patients with DED and 28 eyes of 23 healthy controls were included for examination in this study. The evaluation included the application of an Ocular Surface Disease Index Questionnaire, Schirmer Basic Secretion Test, and anterior OCTA targeting the temporal conjunctiva. AngioTool software was used to quantify the total vessel length and vessel density in the 3 × 3 mm temporal region of interest.

**Results:**

Blood vessel density measurements were compared across the OCTA systems. The total vessel length within the conjunctiva of the DED group (4799.34 ± 834.36) exceeded that of the control eye (3864.89 ± 1455.70) group (*P* < 0.05). However, the difference in vessel density between the two groups was not statistically significant.

**Conclusion:**

Measurement and analysis of conjunctival blood vessels using OCTA exhibited robust repeatability. In dry eyes, the total number of conjunctival blood vessels increased in accordance with disease severity. Hypoxia of conjunctival tissue may be an important cause of dry eye disease.

## 1. Introduction

Dry eye disease (DED) is a common condition characterized by tear film instability [1, 2], manifested as aberrations in tear fluid quality and quantity, which leads to ocular discomfort and visual dysfunction. At present, the worldwide prevalence of DED ranges from 5.5% to 33.7% [3], with the highest incidence observed among females, the elderly, and individuals of Asian descent. The pathogenesis of DED is multifaceted, encompassing a complex interplay involving environmental factors, age, chronic inflammation, and immune dysregulation [4–6]. Elevation in numerous inflammatory factors has been evidenced in the tear fluid [7], showing correlation with an increase in tear osmotic pressure, tear film instability, alterations in tear composition, and corneal injury [8].

Inflammation often culminates in tissue congestion; however, research on the quantitative assessment of conjunctival vasculature in DED remains limited. On the one hand, the relatively low resolution of color images captured by conventional cameras escalates the difficulty of precise vascular analysis. On the other hand, although fluorescein angiography yields superior resolution, its invasive nature renders it impractical for repeat measurements. In this study, we aimed to evaluate the reproducibility of optical coherence tomography angiography (OCTA) for visualizing conjunctival vessels and compared the differences in conjunctival vascular length and density between normal eyes and those afflicted by DED.

## 2. Materials and Methods

This cross-sectional study included patients diagnosed with DED and healthy controls. The study was conducted at the Department of Ophthalmology, First People's Hospital of Suqian City, China, from April to June 2021. The trial conformed to the guidelines outlined in the Declaration of Helsinki and was approved by the Institutional Ethics Committee of the Suqian First People's Hospital. All patients provided written informed consent and underwent a comprehensive ophthalmological evaluation, including visual acuity examination, intraocular pressure measurement, and assessment of the anterior and posterior segments of the eye.

A total of 23 patients with DED and 28 healthy controls were enrolled in this study. The diagnostic criteria included the following: (1) subjective symptoms (mandatory), namely, dryness, foreign body sensation, fatigue, and discomfort; (2) tear film instability (mandatory): break-up time (BUT) <10 s was considered positive and 5 s was considered strongly positive; (3) reduced tear secretion: a lacrimal river height <1 mm; within 5 min of the Schirme I test, a wetted length of filter paper <10 mm was considered positive and less than 5 mm was considered strongly positive; (4) ocular surface damage (enhanced diagnosis): assessment through fluorescein, tiger red, and lisamine green staining; and (5) increased tear osmolality or decreased lactoferrin levels (enhanced diagnosis). The exclusion criteria encompassed patients who exhibited (1) conjunctivitis, keratitis, or other ocular surface inflammatory diseases; (2) a history of eye trauma or eye surgery within the past 3 months; (3) use of contact lenses in the past 3 months; (4) best-corrected visual acuity <20/20; (5) presence of malignant tumors or rheumatic immune diseases; and (6) pterygium or palpebral fissures.

For OCTA imaging, AngioVue (version 2017.1.0.151; Optovue, California, USA) was used to perform split-spectrum amplitude-decorrelated angiography. The OCTA Corneal Adapter module was utilized to scan nasal and temporal conjunctival vessels. All OCTA scans were performed by a single experienced operator.

During the scan of the nasal conjunctival vessels, participants focused on fixed points on the temporal aspect of the eye. Conversely, during the scan of the temporal conjunctival blood vessels, participants focused on fixed points on the nasal side of the eye. The regions of interest were fully exposed during the examination. The scanning area comprised solely of 3×3 mm sections of conjunctival or marginal vessels.

The conjunctival vascular depth hierarchy was manually adjusted, setting a reference depth of 1,200 *μ*m to exclude underlying scleral vessels. Images with a signal strength index below 50, those exhibiting severe artifacts due to poor fixation, and images displaying eyelash occlusion were excluded from the analysis.

**Figure 1:**
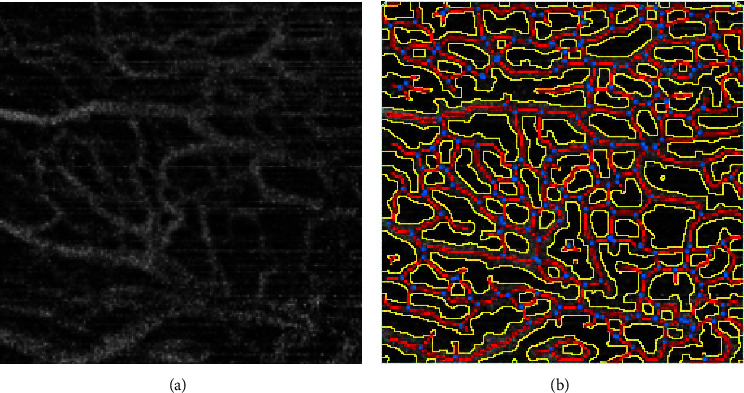
Representative images of conjunctival blood vessels. (a) The area of interest for optical coherence tomography angiography (OCTA) scanning is delineated in the box on the left. The OCTA scan area is 6 × 6 mm after tangential alignment with the corneal limbus, with a subsequent selection of a clear 3 × 3 mm region to ensure precise vascular analysis. (b) Aristotle processed pictures following image manipulation. These images exhibit distinctly marked vascular networks, alongside error identification aimed at discerning pseudoshadows from conjunctival vascular networks.

Raw and captured OCTA images obtained using AngioVue are shown in Figure 1(a). The original OCTA image manifests artifacts along the horizontal axis. The vascular skeleton was isolated from the vascular network, and subsequent morphological manipulation was performed using a vascular tool, as depicted in Figure 1(b). This manipulation yielded a distinct vascular skeleton pattern; however, the blood vessels extracted using this traditional approach exhibited greater thickness compared to actual blood vessels, consequently influencing the accuracy of quantitative blood flow and vessel thickness assessments. Furthermore, the presence of high-noise levels in the original image hampers the capacity of angiotensin to eradicate artifacts and image noise. Consequently, the derived vascular network appears larger than the authentic container, thus reducing the accuracy of container thickness and length determinations. Therefore, to minimize the impact of artifacts on outcomes, we targeted participants aged between 18 and 50 years, as this demographic exhibited higher levels of cooperation and maintained fixation more effectively. All examinations were conducted between 9 and 11 o'clock to avoid possible diurnal variations. In addition, it was noted that individuals afflicted with DED exhibited reduced visual endurance and diminished clarity in the OCTA images, leading to the exclusion of data of many participants.

### 2.1. Statistical Analysis

Statistical analyses were performed using SPSS (version 25.0; SPSS Inc., Chicago, IL, USA). All continuous variables are expressed as means ± standard deviations (means ± SDs). An independent *t*-test was employed for the statistical analysis of continuous variables. All data were subjected to the unpaired samples *t*-test (Welch correction for unqualified variables); *P* < 0.05 was considered statistically significant. Pearson's correlation coefficient was used to analyze the associations between different parameters.

## 3. Results

The study included 7 male and 11 female participants in the DED group, along with 11 males and 12 females in the control group. No significant statistical differences were observed between the two groups with respect to sex or age (Table 1). Patients with diabetes or a history of systemic medication, which may have affected experimental accuracy, were excluded. A subset of participants used frame glasses (22% in the DED group and 11.3% in the control group), while those who used contact lenses were omitted from the study. Owing to the constraints of sample size, distinctions in sex, occupation, and working hours were not taken into account.

All patients underwent assessment using Schirmer's I trial to obtain a dry-eye score, along with an evaluation of dry eye severity through the Ocular Surface Disease Index Questionnaire Dry Eye Rating Scale. Tear film BUT was measured using Whatman 41 filter paper, while corneal health was assessed via a slit lamp biological microscope equipped with a 2% fluorescein and cobalt blue filter. Notably, BUT values in the DED group were significantly shorter than those in the control group. Moreover, Schirmer's degree scores were significantly lower in the DED group than in the control group (Table 2).

The total length of blood vessels in the DED group surpassed that observed in the control group (*P* < 0.05), whereas the vessel density exhibited no statistically significant variance (Table 3).

## 4. Discussion

DED, a prevalent ocular surface disorder, is characterized by tear film instability and/or ocular surface injury due to abnormalities in tear fluid quantity or quality and perturbations in fluid dynamics, accompanied by an increase in tear osmotic pressure [9, 10]. Ocular surface inflammation is thought to be an important factor in the pathogenesis of DED [11–15]. A myriad of intricate interactions among immune and neurogenic inflammatory factors can give rise to ocular epithelial damage, changes in the lower basal plexus density, and exacerbation of DED symptoms [16, 17].

This study ventured into quantitative analysis of conjunctival vessels in patients with DED using OCTA, marking a significant advance beyond previous studies based on mediator-mediated DED research. The revelation of increased conjunctival vessel length among patients with DED evaded elucidation in prior research. Changes in the microvasculature of the superior conjunctival membrane are discerned from our investigation. Notably, we observed no significant increase in the vascular drainage area; however, the length of blood vessels exhibited a significant alteration. Hemodynamic variations potentially underpin the etiology of DED. Understandably, the elongation of blood vessel length prolongs the time that individual red blood cells stay on the ocular surface, although the total count of oxygenated red blood cells does not increase. Moreover, the proliferation and contraction of hypoxic bulbar conjunctival vessels may be an important contributor to the pathogenesis of DED. This insight could yield comprehension into diabetic ocular surface disorders, especially considering the increase in the number of afflicted patients with DED. In addition, the occurrence of narrow eye fissures and transient irregularities of activity in patients with DED culminates in hypoxic activity of the bulbar conjunctiva upon exposure to air. Consequently, the capability of the bulbar conjunctiva to reoxygenate is reduced.

Inflammatory mediators are recognized for their capacity to stimulate alterations within the microenvironment, increasing local blood flow, and promoting immune cell activation. Whether the reduction in blood supply instigates further impairment of the functionality of ocular surface cells requires further investigation. Notably, an evident correlation emerges between shifts in the conjunctival vascular length and the severity of DED. Inflammation activates various angiogenic factors, thereby disrupting the natural equilibrium of the conjunctival epithelial cell microenvironment and accelerating the formation of new blood vessels. This mechanism may explain why dry eye conjunctival vascularity increases under such conditions. However, to date, comprehensive studies evaluating conjunctival vessels in patients with DED have been limited. This study capitalizes on OCTA as an eminently reproducible tool for detecting conjunctival vessels, enabling noncontact scanning in seconds [18–20]^.^

As alterations manifest in the length of conjunctival vessels, the time required for the activation and residence of inflammatory factors traversing the ocular surface increases significantly, affecting the function of conjunctival goblet cells and related immune cells. This study demonstrated the potential utility of OCTA in the context of DED encompassing parameters such as the length and density of conjunctival vessels. However, further research is needed to validate the effectiveness of this tool, not only for guiding individuals with DED toward appropriate treatment options but also for addressing the limitations at hand. It is pertinent to acknowledge that OCTA cannot detect the minimal blood flow within all vessels or signals occluded by corneal opacity. Furthermore, it cannot visualize vessel leakage or the direction of blood flow. These limitations potentially affect the analysis of ocular surface blood flow.

## 5. Conclusion

Our findings demonstrated the superior sensitivity and reproducibility of OCTA in the quantification of conjunctival vessels. Patients with DED exhibit extended total conjunctival vessel lengths along with an abundance of blood vessels. The interplay of hypoxia and inflammatory factors may contribute to this phenomenon. Moreover, OCTA-derived measurements of conjunctival vessels in DED provide an objective basis for assessing the severity of the condition. It is crucial to recognize that substantial work remains ahead to better elucidate the interactions between these factors in chronic DED.

## Figures and Tables

**Table 1:** Comparison of general conditions between the DED and control groups.

Parameters	DED group	Control group	*P*
Number (eye)	23	28	—
Age (y)	35.87 ± 8.90	37.46 ± 10.18	0.56
Sex: male/female	7/11	11/12	—
Daily electronic-use time (h)	3.55 ± 1.26	1.48 ± 0.69	0.01

DED, dry eye disease.

**Table 2:** BUT, OSDI, and Schirmer's I examinations in the DED and control groups.

Parameters	DED group	Control group	*P*
Number (eye)	23	28	—
BUT (s)	3.70 ± 1.72	11.07 ± 2.14	0.01
OSDI	36.48 ± 5.98	17.43 ± 3.57	0.01
Schirmer's I (mm)	4.78 ± 2.37	16.64 ± 5.23	0.01

DED, dry eye disease; BUT, break-up time; OSDI, ocular surface disease index.

**Table 3:** Total conjunctival vascular length, vessels area, and vascular density in the DED and control groups.

Parameters	DED group	Control group	*P*
Number (eye)	23	28	—
Total length of blood vessels	4799.34 ± 834.36	3864.89 ± 1455.70	0.01
Vessel area	32126.69 ± 4284.41	31516.82 ± 5090.21	0.65
Vessel density	0.518 ± 0.0675	0.509 ± 0.0813	0.70

DED, dry eye disease.

## Data Availability

The relevant data that support the findings of the study are available from the corresponding author upon reasonable request.

## Abbreviations

OCTA: Optical coherence tomography angiography
DED: Dry eye disease.
